# Comprehensive computational analysis via Adverse Outcome Pathways and Aggregate Exposure Pathways in exploring synergistic effects from radon and tobacco smoke on lung cancer

**DOI:** 10.3389/fpubh.2025.1571290

**Published:** 2025-07-31

**Authors:** Thomas Jaylet, Vinita Chauhan, Laura Mezquita, Nadia Boroumand, Olivier Laurent, Karine Elihn, Lovisa Lundholm, Olivier Armant, Karine Audouze

**Affiliations:** ^1^Université Paris Cité, Inserm, HealthFex, Paris, France; ^2^Consumer and Clinical Radiation Protection Bureau, Health Canada, Ottawa, ON, Canada; ^3^Medical Oncology Department, Hospital Clínic of Barcelona; Laboratory of Translational Genomics and Targeted Therapies in Solid Tumors, IDIBAPS; Department of Medicine, University of Barcelona, Barcelona, Spain; ^4^Centre for Radiation Protection Research, Department of Molecular Biosciences, The Wenner-Gren Institute, Stockholm University, Stockholm, Spain; ^5^PSE-SANTE/SESANE/LEPID, Autorité de Sûreté Nucléaire et de Radioprotection (ASNR), Fontenay-Aux-Roses, France; ^6^Department of Environmental Science, Stockholm University, Stockholm, Sweden; ^7^PSE-ENV/SERPEN/LECO, Autorité de Sûreté Nucléaire et de Radioprotection (ASNR), Saint-Paul-Lez-Durance, Cadarache, France

**Keywords:** Aggregate Exposure Pathway (AEP), Adverse Outcome Pathways (AOP), radon, tobacco smoke, lung cancer, computational toxicology, text mining, AOP-helpFinder

## Abstract

Lung cancer remains the leading cause of cancer mortality worldwide, with tobacco smoke and radon exposure being the primary risk factors. The interaction between these two factors has been described as sub-multiplicative, but a better understanding is needed of how they jointly contribute to lung carcinogenesis. In this context, a comprehensive analysis of current knowledge regarding the effects of radon and tobacco smoke on lung cancer was conducted using a computational approach. Information on this co-exposure was extracted and clustered from databases, particularly the literature, using the text mining tool AOP-helpFinder and other artificial intelligence (AI) resources. The collected information was then organized into Aggregate Exposure Pathway (AEP) and Adverse Outcome Pathways (AOP) models. AEPs and AOPs represent analytical concepts useful for assessing the potential risks associated with exposure to various stressors. AOPs provide a structured framework to organize knowledge of essential Key Events (KEs) from a Molecular Initiating Event (MIE) to an Adverse Outcome (AO) at an organism or population level, while AEPs model exposures from the initial source of the stressor to the internal exposure site within the target organism, situated upstream of the AOP. Combining these frameworks offered an integrated method for knowledge consolidation of radon and tobacco smoke, detailing the association from the environment to a mechanistic level, and highlighting specific differences between the two stressors in DNA damage, mutational profiles, and histological types. This approach also identified gaps in understanding joint exposure, particularly the lack of mechanistic studies on the precise role of certain KEs such as inflammation, as well as the need for studies that more closely replicate real-world exposure conditions. In conclusion, this study demonstrates the potential of AI and machine learning tools in developing alternative toxicological models. It highlights the complex interaction between radon and tobacco smoke and encourages collaboration among scientific communities to conduct future studies aiming to fully understand the mechanisms associated with this co-exposure.

## 1 Introduction

### 1.1 Context

Lung cancer is among the most frequently diagnosed cancers and represents the leading cause of cancer death (1). In 2022, it accounted for nearly 2.5 million new cases, representing about one in eight cancers worldwide (12.4%), and approximately 1.8 million deaths, accounting for 18.7% of total cancer deaths (1). In the landscape of environmental risk factors, tobacco smoke and radon stand out prominently (2).

Tobacco smoke contain over 7,000 chemical compounds, of which approximately 250 chemicals possess toxicological properties and nearly 70 of them are well-established carcinogens (3, 4). Key toxic substances in tobacco smoke include carbon monoxide, benzene, formaldehyde, polycyclic aromatic hydrocarbons (PAHs), and nitrosamines (5), all of which are linked to cancer development. Additionally, tobacco contains naturally occurring radionuclides such as polonium-210 (5, 6), as well as heavy metals like lead, cadmium, and arsenic, which can accumulate in the body, leading to significant health issues (7). The combustion of tobacco products, such as cigarettes, cigars, and pipe tobacco, is the primary source of exposure to these compounds. With a global smoking population surpassing one billion, this exposure to harmful tobacco chemicals has become ubiquitous (8). The International Agency for Research on Cancer (IARC, part of the World Health Organization–WHO) officially classified tobacco smoke as a carcinogen in 1986 (9–11). This complex mixture of toxic substances makes tobacco smoke the leading carcinogen in lung cancer development, responsible for approximately 85% of cases (12). The associated risk increases substantially, being five times higher in light smokers (1–4 cigarettes per day) and exceeding twenty times in heavy smokers (more than 20 cigarettes per day) (13, 14).

Alongside smoking, radon exposure also poses a significant threat (15). Radon is formed by the decay of uranium-238 and is present in the Earth’s crust, with heightened prevalence in geological formations rich in uranium, including large granite massifs, certain sandstones, and black shales. In the outdoor environments, radon typically dissipates into the air, typically rendering it harmless. However, in confined spaces such as underground mines, caves, and buildings where radon can infiltrate through various pathways, including cracks, openings in the foundations, and plumbing, leading to accumulation and occasionally reaching high concentrations (16). Radon was classified as a human carcinogen by the IARC in 1988 (17), and residential radon exposure has been shown to significantly contribute to lung cancer development through the emission of alpha particles that directly damage lung cells (13, 18). Epidemiological evidence strongly supports this association, with extensive documentation from pooled cohort and nested case–control studies among radon-exposed miners (19–22), as well as from landmark case–control studies in the general population. Collaborative research pooling 13 case–control studies from Europe and 7 from North America has notably demonstrated a significant association between long-term exposure to residential radon and an increased risk of developing lung cancer (13, 23). According to these case–control studies in the general population, the initial risk of lung cancer increases by approximately 16% for each 100 Bq/m^3^ increase in residential radon concentration (13). The WHO recommends a radon level below 100 Bq/m^3^ (15), and the European Directive 59/2013 has established a reference level of 300 Bq/m^3^ for homes and workplaces, which should not be exceeded (24). At the European level, several regions exceed these recommendations. For example, some French departments rich in granite massifs report levels above 200 Bq/m^3^ (25). Similarly, some Northern European countries, such as Sweden and Finland, report high radon concentrations, with over 330,000 Swedish single-family houses exceeding 200 Bq/m^3^ and certain Finnish public buildings, including schools, sometimes surpassing 300 Bq/m^3^ (26, 27). According to the WHO, radon is responsible for 3 to 14% of lung cancer cases, depending on the national average radon level and smoking prevalence in a country, making it the second leading cause of lung cancer among tobacco smokers and one of the leading causes among non-smokers (28).

Combined exposure to radon and tobacco smoke has been shown to significantly increase the risk of lung cancer compared to exposure to either factor alone (29), with increasing radon levels and longer smoking durations both associated with higher incidence rates (30). Considering risk prediction in the population, analyses of miner data suggest that the observed outcome is best described by a sub-multiplicative relationship. This type of interaction is defined according to current terminology, which includes additive, sub-additive, sub-multiplicative, and multiplicative effects. In this context, a sub-multiplicative interaction refers to a joint effect that is greater than additive but less than multiplicative, as defined by Laurier et al. and UNSCEAR (2019, Annex B) (31, 32). This synergy may also be relevant for individuals exposed to secondhand smoke (33).

Independently, both radon and tobacco smoke have been shown to induce oxidative stress and DNA damage in lung cells (18, 34). When combined, they may potentiate each other’s effects through complex biological interactions that promote the initiation and progression of lung cancer, thereby contributing to the observed sub-multiplicative risk pattern (22, 33). Interestingly, some epidemiological and meta-analytic studies have suggested exposure-dependent differences in lung cancer histology, with small cell lung carcinoma (SCLC) more frequently associated with radon exposure in smokers, and adenocarcinoma more common in individuals with lower tobacco exposure (35). While these findings indicate potential subtype-specific associations, no conclusive mechanistic distinction has been established, and the way in which radon and tobacco smoke interact and jointly contribute to lung carcinogenesis remains incompletely understood.

In this context, the present article aims to explore current knowledge regarding the effects of radon and tobacco smoke on lung cancer, in order to identify possible pathways that could better model the synergies during co-exposure, while highlighting the gaps in our understanding of their interactions as combined mechanisms. Clarifying these aspects is not only essential for developing more effective prevention strategies, but it could also provide a solid foundation for future scientific research on lung carcinogenesis. To address this complex issue from both an exposure and a mechanistic perspective, this study builds on two key analytical frameworks, the Adverse Outcome Pathway (AOP) and the Aggregate Exposure Pathway (AEP) frameworks.

### 1.2 Adverse Outcome Pathways and Aggregate Exposure Pathways

The AOP offers a structured framework to illustrate the underlying biological mechanisms, connecting a molecular initiating event (MIE) through a series of key events (KE) up to the occurrence of an adverse outcome (AO) at the organism or population level (36). All the connections between these events are described by Key Event Relationships (KERs), which are defined based on scientific knowledge that describes and supports these relationships. The AOP framework has already been used to describe the disruption of biological pathways resulting from exposure to ionizing radiation (IR) or tobacco smoke. In the context of lung cancer, AOP 272 describes the damage and mutations resulting from exposure to IRs (18). For tobacco smoke, although there is currently no AOP directly related to lung cancer, there are AOPs linking tobacco smoke to reductions in lung function via oxidative stress (AOP 411, 424, and 425), as well as AOPs describing the link between exposure to PAHs, compounds present in tobacco smoke, and the formation of DNA adducts and mutations.^1^ This knowledge can be further consolidated by organizing these pathways into an AOP network (AOPN), which enables the integration of mechanistic information related to different stressors within a single, structured framework. AOPNs are particularly well suited for representing complex biological responses to real life exposures involving multiple agents, as they allow for the identification of pathway convergence, divergence, and interconnections. In the context of co-exposure to tobacco smoke and radon gas, this approach helps highlight shared or interacting key events and supports a more comprehensive understanding of the mechanistic processes involved in lung carcinogenesis.

AEPs are built on a conceptual framework similar to AOPs. While AOPs provide a structured representation of biological mechanisms from an MIE to an AO, AEPs describe the progression of a stressor from its environmental source to the site of internal exposure, situated upstream of the AOP’s MIE (37). Together, these frameworks allow for a multidimensional representation of how environmental factors, such as tobacco smoke and radon, contribute to disease development. This perspective is consistent with recent efforts to connect exposure science and toxicology by integrating AEP and AOP frameworks, in order to better capture the complexity of chemical interactions across the full source-to-effect pathway (38). It also reflects the growing interest in applying mechanistic strategies to investigate the effects of chemical mixtures on cancer development (39). Following this direction, recent developments have shown the value of computational approaches to support the construction of such integrative models. In particular, text mining has proven effective for rapidly extracting bibliographic evidence from extensive scientific literature. At the same time, omics-based data are increasingly used to identify KEs and reinforce the scientific basis for evaluating AOPs through weight-of-evidence assessments (40–43).

Here, we propose the first fully computational workflow that combines automated literature mining and machine learning with established AOP and AEP frameworks to investigate how co-exposure to radon and tobacco smoke contributes to lung cancer. The approach integrates data from expert-curated resources (AOP-Wiki, CTD, GeneCards, DisGeNET), archival datasets (StoreDB), and scientific literature (PubMed), enabling the construction of a comprehensive source-to-effect representation. Exposure-related information was used to inform the AEP component, while biological and molecular data supported the development of an AOPN. This integrative framework offers a structured means to explore their combined effects, identify knowledge gaps, and support the mechanistic interpretation of pathways involved in lung cancer development.

## 2 Materials and methods

### 2.1 Automatic literature screening: extraction of a corpus of scientific articles

#### 2.1.1 Databases description

To start, a comprehensive screening of various databases was conducted to gather a collection of keywords representing biological events involved in, or related to lung cancer, which would be pertinent for the creation of our AEP/ AOPN. These keywords include MIEs (such as the initial energy deposition, receptor or gene activation, and other molecular occurrences), KEs (such as cellular damage, specific gene mutations, and alterations in biological pathways), as well as keywords directly related to AOs (such as the different histological types of lung cancer). The AOP-Wiki, a community reference database for AOPs^2^, served as a key resource in this study. It granted access to prior knowledge and insights into existing AOPs potentially disrupted by these stressors, facilitating the collection of essential information on biological events (MIEs, KEs) affected by IRs and tobacco (e.g., oxidative stress, DNA damage, mutations). Additionally, databases such as the CTD, which highlights the health impacts of toxic agents and provides insights into the relationships between stressors and disease^3^, GeneCards, an integrative resource containing information about human genes from various online sources^4^, and DisGeNET, focused on compiling data regarding the associations between genes and human diseases^5^, were screened to extract a set of AO (e.g., small cell lung cancer, non-small cell lung cancer) and altered genes (e.g., *TP53*, *KRAS*, *EGFR*) related to lung cancer.

**Figure 1:**
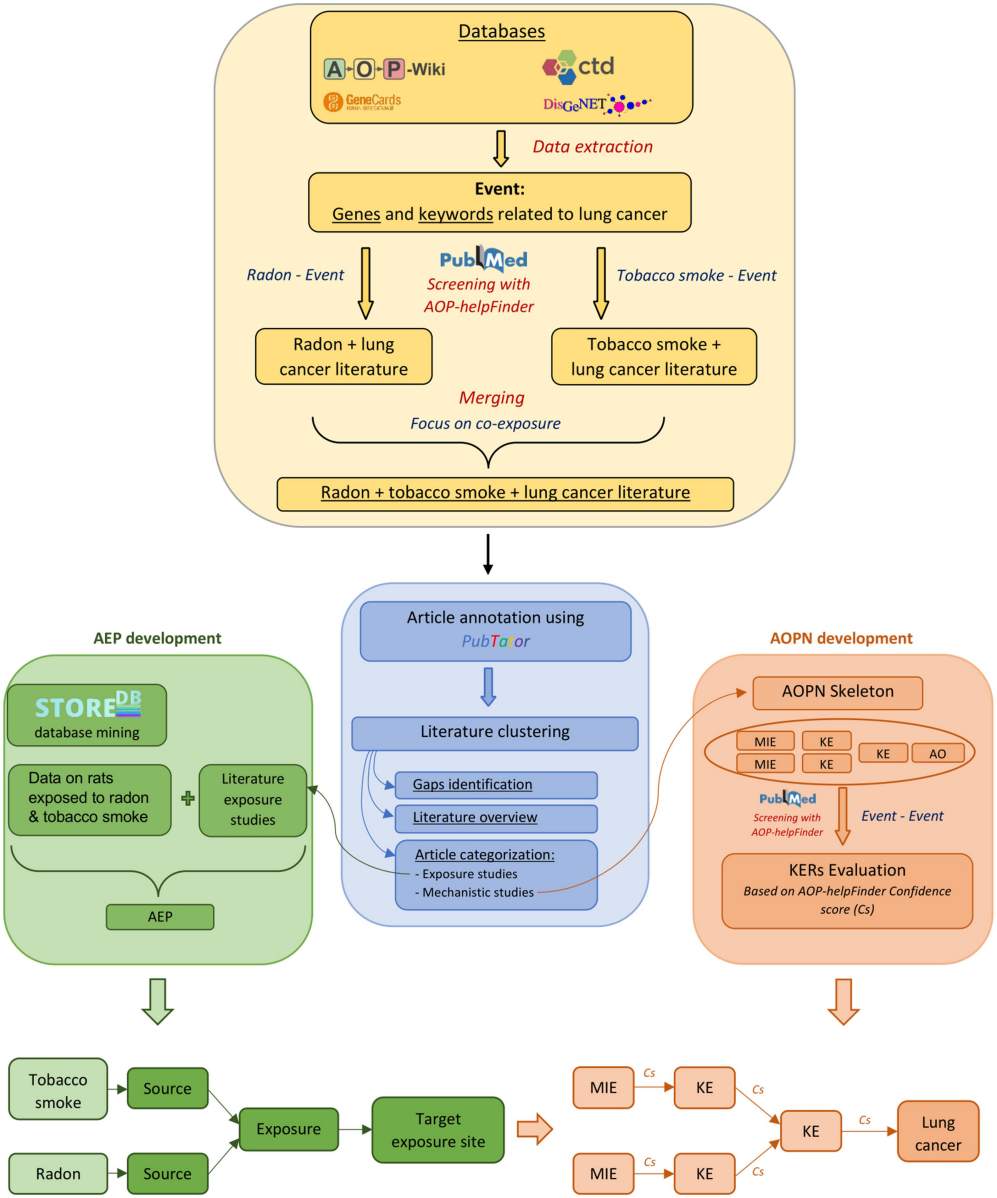
Overview of the study progression. The development of the AOPN and AEP is based on information derived from scientific literature and exposure data, obtained through databases and bioinformatics tools. The identification of gaps and the overview of the literature following clustering involve examining and categorizing the available data and types of data. This includes assessing the number of mechanistic vs. epidemiological studies, or identifying which KEs are most and least studied in the context of radon-tobacco co-exposure. Studies focusing on exposure will be preferentially used for the development of the AEP, while studies focusing on mechanistic relationships will be used for the development of the AOPN. * The term “Event” in the Stressor-Event search corresponds to the 238 biological keywords associated with lung cancer, while in the Event-Event search, it refers exclusively to the final components (MIE, KE, AO) included in the AOPN for evaluating KERs.

In total, a set of 238 keywords (e.g., biological events associated with lung cancer) were extracted from these databases. This list of keywords, referenced in Supplementary Table S1, was used for the automated screening of the literature with AOP-helpFinder, establishing a solid scientific corpus for the modeling and assessment of AEP and AOPN models focused on the combined effects of radon and tobacco smoke (Figure 1).

#### 2.1.2 Automatic literature screening using AOP-helpFinder

AOP-helpFinder is an artificial intelligence (AI)-powered text mining tool that screens abstracts on PubMed to extract associations between stressors and biological events (stressor-event module), or between biological events (event-event module) provided as input by a user. Based on graph theory, the tool identifies relevant relationships within these abstracts, thereby facilitating the compilation of sparse information from the literature and enabling the organization of a pertinent scientific literature dataset (44–46). In this study, we initially used the stressor-event module of AOP-helpFinder to extract all published information on the relationship between tobacco smoke and the 238 lung cancer-related keywords extracted from databases (see section 2.1.1 and Supplementary Table S1), as well as radon and these same keywords. Therefore, two independent runs of the tool were executed. The first run, aimed at identifying relationships between radon and the biological keywords, identified 1,571 relevant articles from the 9,308 radon-related articles available on PubMed (02/2024), meaning articles containing at least one association between radon and one of the 238 keywords on the list. The second run, focusing on tobacco smoke, identified 38,198 articles with relevant links among the 246,988 tobacco-related articles reviewed (02/2024) (Figure 1). Given our goal to investigate the joint effects of radon and tobacco smoke, the results from both runs were merged, keeping only articles found in both searches. From this merge, 378 relevant articles were identified (Supplementary Table S2). Thus, this dataset provides a scientific basis for constructing models of AEPs and AOPN, facilitating a comprehensive review of the literature on the combined effects of tobacco smoke and radon exposure, with the aim of identifying common and potentially interacting mechanisms between both stressors.

### 2.2 Literature clustering

#### 2.2.1 Automatic article annotation using PubTator

To complement the information extracted by AOP-helpFinder, PubTator (version 2) was employed on our dataset of 378 articles, enabling the capture of additional details. PubTator is an automatic biomedical text annotation tool used to extract relevant information from scientific articles. It identifies and tags bioconcepts, such as genes, diseases, and chemical substances, to facilitate research and data analysis in the biomedical field (47).

Following this, each article in our dataset is annotated with the lung cancer-related event identified by AOP-helpFinder, its corresponding event class (e.g., MIE, KE, AO, manually assigned to each event), and the studied model or species (human, animal models, or cellular models) identified by PubTator. The entire annotated dataset is available in Supplementary Table S2.

#### 2.2.2 Clustering

The annotated dataset described above was used for unsupervised clustering with the k-means algorithm. Each article was represented using key categorical variables, including the manually assigned event class (MIE, KE, AO), as well as the experimental model, species, and exposure context extracted via PubTator (e.g., “human,” “animals,” “cells,” or “miner”) (Supplementary Table S2). These variables were concatenated into a single string and vectorized using a Term Frequency–Inverse Document Frequency (TF-IDF) transformation, resulting in a sparse feature matrix.

**Figure 2:**
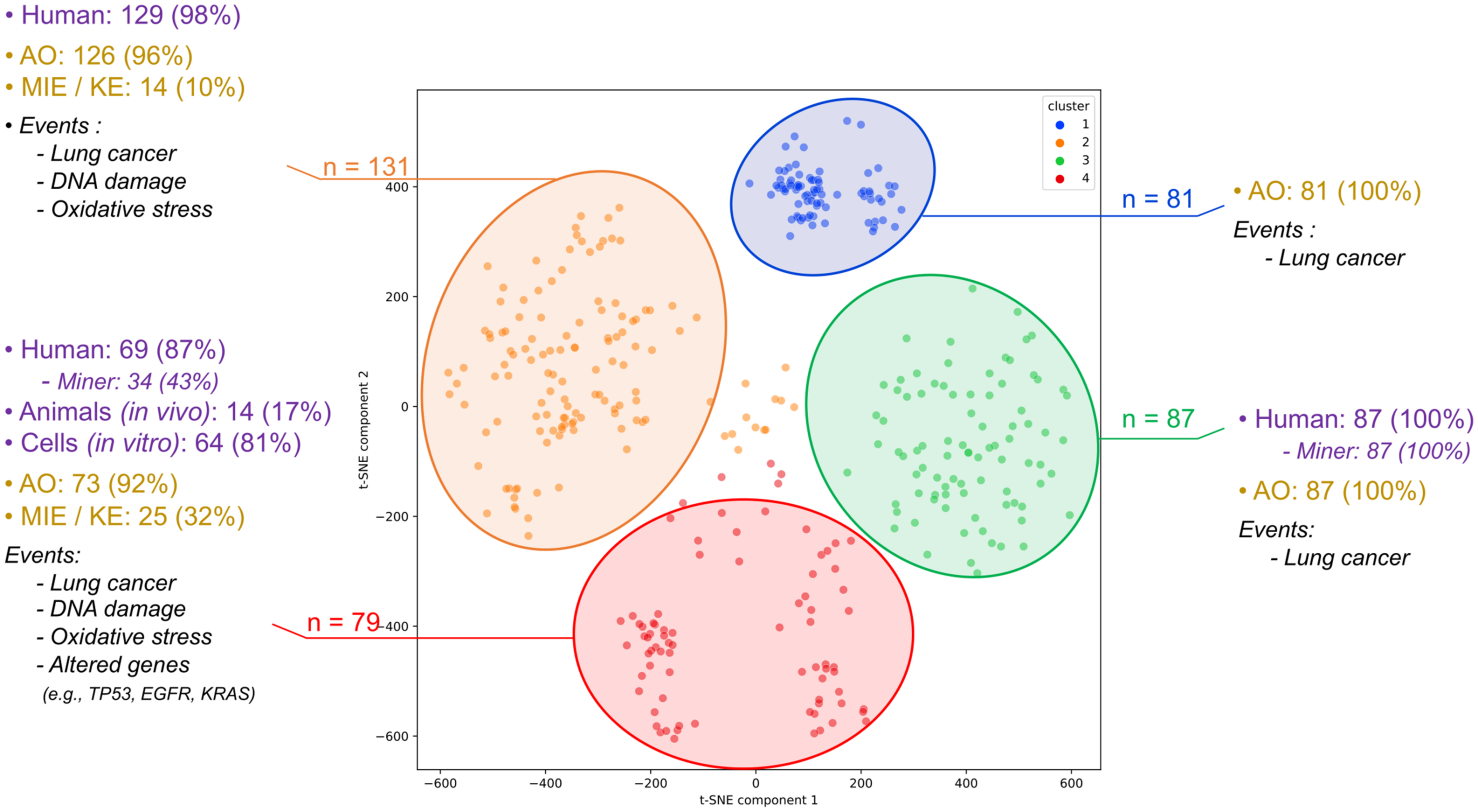
Clustering of the 378 articles assessing the combined impact of radon and tobacco on lung cancer. This classification was carried out into 4 clusters using the k-means method. Each cluster is detailed by the types of studies conducted (including human, animal model, and cellular research) and displays the type and predominant biological events identified within each cluster.

To reduce dimensionality and structure the data prior to clustering, a t-distributed Stochastic Neighbor Embedding (t-SNE) was applied to the TF-IDF matrix, producing two-dimensional embeddings. These embeddings served as input for the k-means clustering algorithm. The optimal number of clusters (k) was determined using the elbow method, which involved plotting the sum of squared errors (SSE) for values of k ranging from 2 to 9. The resulting curve (Supplementary Figure S1) showed a clear inflection point at k = 4, which was selected as the optimal number of clusters. K-means clustering was then performed using the “scikit-learn” Python library (random_state = 42, n_init = 10) to ensure reproducibility and robustness. The resulting clusters were visualized in a t-SNE scatterplot (Figure 2).

Each cluster was analyzed to identify distinguishing features, enabling us to differentiate between groups of epidemiological studies (e.g., case–control studies in miners), mechanistic studies using animal or cellular models, and other combinations. Full cluster details are provided in Supplementary Table S2. This clustering step provides an automated overview of the literature and highlights potential gaps in specific data types. It also enables the prioritization and identification of article groups, facilitating the development of AOPN and AEP models. In particular, mechanistic studies are prioritized for AOPN construction, while exposure-related studies are targeted for AEP development.

### 2.3 AEP development combining literature and StoreDB screening

The AEP development is guided by an analysis of studies examining radon and tobacco smoke exposure, drawing from a dataset of 378 annotated articles. In addition to evidence from the literature, data from rodent studies exposed to various doses of radon and/or tobacco smoke stored in StoreDB were compiled and analyzed. StoreDB, established under the EC Euratom program, serves as an archival and sharing platform for data from radiation research, encouraging collaboration within the research community in this field. From this database, three relevant studies were extracted: study no.1047 (48), study no. 1050 (49), and study no. 1051 (50). Exposure was expressed in Working Level Months (WLM), the standard unit used in epidemiological studies on radon exposure among mine workers, whose data have substantially contributed to the understanding of radon-related lung cancer risk. The WLM represents cumulative exposure over a standard work month of 170 h, with radon progeny concentration measured in Working Levels (WL). For interpretability, 1 WLM corresponds to exposure to 3,700 Bq/m^3^ of radon progeny for 170 h, or approximately one year of residential exposure to 230 Bq/m^3^, which is equivalent to ~5 mSv effective dose, according to ICRP Publication 137 (51).

Study no.1047 investigated the effects of different radon doses, expressed in WLM, and dose rates, expressed in WL, on Sprague–Dawley rats continuously exposed over a period of up to 3 months. Study no.1051 aimed to determine the risks of lung tumors after inhalation of radon with tobacco smoke. In this study, a cohort of Sprague–Dawley rats was subjected to high doses (1,600 or 1800 WLM) of radon with or without tobacco smoke exposure. Study no.1050 evaluated the risks of tumors after radon inhalation. In this study, 500 rats were exposed to relatively moderate doses (25 WLM) at an average dose rate (150 WL). Thus, the analysis of data from both experimental studies contained in StoreDB and studies from the literature on radon and tobacco smoke exposure, provides an integrated illustration of the various pathways through which radon and tobacco smoke may come into contact with the lungs and how this translates to lung cancer risk. This integrated illustration is provided by the AEP framework, offering insight into the synergistic effects upon exposure to evaluate health risks.

### 2.4 AOPN development

#### 2.4.1 Structuring of the AOPN skeleton

Based on the examination of articles contained in the dataset (Supplementary Table S2), an AOPN focusing on the mechanisms common to both radon and tobacco smoke in the context of lung cancer was developed. This AOPN illustrates the biological pathways and events that are simultaneously disrupted by these two stressors. With the KEs and the AO selected to highlight their shared biological alterations, this framework enhances the understanding of how these diverse stressors converge at similar biological endpoints in lung cancer development. For the common events (KE, AO), the specific characteristics attributable to each stressor were meticulously considered and discussed (e.g., such as mutational profiles and histological forms of lung cancers). Furthermore, given the distinct natures of these factors, e.g., chemical for tobacco smoke and physical for the IR from radon, the MIEs were addressed separately. This distinction allowed for an in-depth examination of the role each factor plays in initiating subsequent damage, thereby providing a comprehensive insight into their individual contributions to the pathology of lung cancer.

#### 2.4.2 Evaluation of the KER using AOP-helpFinder event-event

After identifying all relevant events (MIE, KE, and AO) in the AOPN, the subsequent step involved analyzing and assessing the relationships between these events, referred to as KERs. Traditionally, the weight-of-evidence supporting KERs is assessed using the Bradford-Hill (BH) criteria. This framework evaluates several key aspects: biological plausibility (i.e., whether the upstream and downstream events are mechanistically linked), the essentiality of key events (i.e., whether blocking an upstream KE prevents the occurrence of downstream events), empirical support (i.e., consistency with dose–response, temporal, and incidence patterns), and overall coherence across studies (52). Although robust, this method is time-consuming and requires detailed experimental data, making it more suitable for regulatory applications or the development of quantitative AOPs.

In this study, KERs were evaluated through an automated, literature-based approach using the *event–event* module of AOP-helpFinder. PubMed abstracts were computationally analyzed in a stressor-agnostic manner to identify pairs of biological events from the AOPN that are reported together more frequently than would be expected given their individual frequencies across the database. For each pair of events (MIE to KE, KE to KE, or KE to AO), one-sided Fisher’s exact tests were applied, and the resulting *p*-values were used to assign a Confidence Score (*Cs*), categorized from “Low” to “Very High” (46). This score reflects both the strength of correlation between the events and the level of support found in the scientific literature for the corresponding KER.

Although this approach does not provide the same level of mechanistic detail as the BH criteria, it has already proven effective in offering a structured and reproducible way to explore and prioritize KERs. It therefore represents a scalable and efficient first step to characterize relationships within the AOPN (46). The results of this computational evaluation are summarized in Table 1. To complement this analysis, relevant information on KERs from the AOP-Wiki was also taken into account when available and is presented in the dedicated section.

**Table 1:** Computational evaluation of KERs using AOP-helpFinder.

N° KER	KE leads to KE		Cs	Link
KER 1	Deposition of energy	DNA double-strand breaks (DSB)	★★★★★	734
KER 2	Deposition of energy	Reactive oxygen species (ROS)	★★★★	431
KER 3	AhR activation	CYP activation	★★★★★	82
	CYP activation	DNA adducts (bulky)	★★★★★	25
KER 4	CYP activation	Reactive oxygen species (ROS)	★★★★	32
KER 5	Reactive oxygen species (ROS)	Oxidative DNA damage	★★★★★	1183
KER 6	DNA damage	Inadequate DNA repair (overwhelmed)	★★★	36
KER 7	Inadequate DNA repair (overwhelmed)	Mutations (genetic alterations)	★★★★	68
	DNA damage	Mutations (genetic alterations)	★★★★★	4454
KER 8	Mutations (genetic alterations)	Altered gene expression/protein expression & function	★★★★★	1772
KER 9	Epigenetic changes	Altered gene expression/protein expression & function	★★★★	543
KER 10	Altered gene expression/protein expression & function	Cell cycle alteration/checkpoint failure	★★★★	190
		Decreased apoptosis	★★★	58
		Increased proliferation	★★★	52
	Mutations (genetic alterations)	Cell cycle alteration/checkpoint failure	★★★★	163
		Decreased apoptosis	★★★★	404
		Increased proliferation	★★★★	719
KER 11	Cell cycle alteration/Checkpoint failure	Increased proliferation	★★★★	47
	Increased proliferation	Lung cancer|NSCLC|SCLC	★★★★	275
KER 12	Decreased apoptosis	Lung cancer|NSCLC|SCLC	★★★★	295
Indirect KER (non-adjacent)	Epigenetic changes	DNA damage	★★★★	262
Other relevant non-adjacent KERs	TP53 mutation	Lung cancer|NSCLC|SCLC	★★★★	589
	KRAS mutation		★★★★★	1040
	EGFR mutation		★★★★★	4593
	Mutations (genetic alterations)		★★★★★	12221
	Epigenetic changes		★★★★	611

The table summarizes the correlation associated with each KER identified in the AOPN, as assessed through computational analysis of co-occurrence terms in PubMed abstracts using the *event–event* module of AOP-helpFinder. The Cs reflects how frequently the two events are reported together in the literature and is categorized as follows: [★★★★★] = “very high,” [★★★★] = “high,” and [★★★] = “moderate.” Scores corresponding to “quite low” [★★] and “low” [★] were not found in this dataset. The “Links” column indicates the number of PubMed abstracts in which the two events were co-mentioned. To improve detection and refine scoring, several synonyms were used for each KE during the computational search process, and the full list is provided in Supplementary Table S3.

## 3 Results

### 3.1 Extraction and clustering of literature

**Figure 3:**
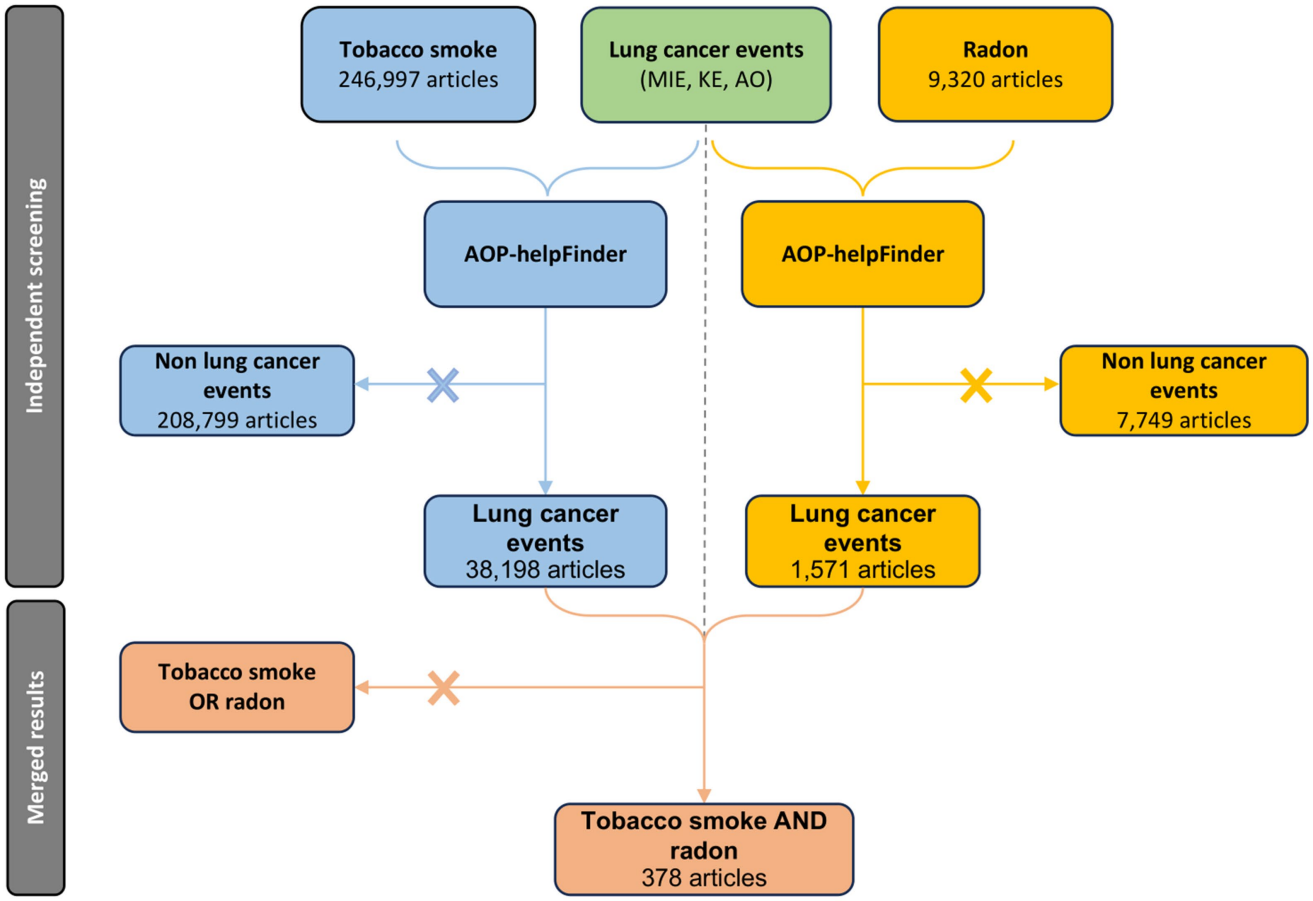
PRISMA diagram of the specific automatic process leading to the acquisition of a relevant article pool for the study of co-exposure to radon and tobacco smoke in the context of lung cancer.

Preliminary searches were conducted with AOP-helpFinder to identify stressor-event pairs separately for radon and tobacco. The search for tobacco identified 38,198 articles investigating the relationship between tobacco smoke and one of our 238 specified lung cancer-related events. The search for radon identified 1,571 articles focusing on radon in relation to these same events (Figure 3). These numbers highlight the significant scientific interest in the two main agents associated with lung cancer, with a notable emphasis on tobacco research. However, when the datasets are combined, only 378 articles explore the synergistic effects of both radon and tobacco smoke (Figure 3). This gap is particularly concerning given that tobacco smoke and radon are the primary risk factors for lung cancer, with widespread exposure among the population.

These 378 studies were then clustered based on annotations obtained with AOP-helpFinder and PubTator (i.e., biological event, species, or study model). Articles were vectorized using TF-IDF, embedded via t-SNE, and grouped using the k-means algorithm into four clusters, with the number of clusters determined using the elbow method (see section 2.2.2 of the Materials and Methods).

Upon analyzing the four clusters derived from the 378 articles, a clear predominance of epidemiological studies involving human populations becomes apparent. Clusters 1, 2, and 3 consist almost exclusively of human studies, with clusters 2 and 3 specifically focusing on cohorts of miners (Figure 2). These clusters are strongly characterized by the presence of AO, predominantly lung cancer and its subtypes (Supplementary Table S2), while providing limited information on upstream biological events. The mechanistic dimension is underrepresented, with few mentions of MIE or KE. In contrast, cluster 4 and to a lesser extent, a portion of cluster 1, includes studies conducted on animal or cellular models and provides more mechanistic insights into the combined effects of radon and tobacco smoke. These articles report KEs such as oxidative stress (e.g., reactive oxygen species), DNA damage (e.g., strand breaks, oxidative lesions, DNA adducts), and altered genes frequently associated with lung tumorigenesis, such as *TP53, KRAS,* and *EGFR*. Some studies also mention epigenetic modifications, including methylation changes, although these remain rare (Supplementary Table S2).

Overall, the four clusters capture distinct domains of the literature: clusters 1–3 are centered around human epidemiological data and AO reporting, while cluster 4 gathers mechanistic studies involving MIEs and KEs. This structuring highlights a gap in integrated mechanistic data, especially regarding the joint functional effects of radon and tobacco. Together, the set of 378 co-exposure articles and the clustering analysis conducted on this dataset provided a structured means to consolidate existing evidence on radon and tobacco interactions and to identify gaps in mechanistic data, thereby supporting the development of the AEP and AOPN conceptual models presented below. The articles from clusters 4 and 2, containing information on biological pathways, will be preferentially used for developing the AOPN, while some of the articles from clusters 1 and 3, notably including co-exposure investigations, will be employed for the development of the AEP.

### 3.2 Construction of AEP model for radon and tobacco

The purpose of the proposed AEP is to identify common exposure pathways between radon and tobacco smoke, and to demonstrate and discuss the influence of each stressor on the other. The aim is to elucidate why co-ocurrent exposure to both stressors leads to a higher, sub-multiplicative incidence of lung cancer in the population (22).

Studies conducted on rats, derived from mining the StoreDB database, were aimed at describing the risks of lung tumors after exposure to radon with or without tobacco smoke, with the goal of better characterizing observations made among uranium miners. Studies no. 1047 and no. 1050 explored the impact of radon alone. Study no. 1047 aimed to evaluate the effects of different doses and dose rates on Sprague–Dawley rats continuously exposed for up to 3 months. It notably revealed a decline in survival for rats exposed to a relatively moderate dose (100 WLM) at high dose rates (48). In study no.1050 (49), 500 rats were exposed to a total dose of 25 WLM at 150 WL, resulting in 14 of them developing cancers. This equates to approximately 3% of cases, a rate that stands notably higher than the global incidence of lung cancer, and higher compared to 0.83% of lung cancer in Sprague Daley rates not exposed to radon (53, 54). Study no. 1051 goes further, exploring exposure to radon alone, as well as combined with tobacco smoke. Exposing 50 male Sprague–Dawley rats to a radon exposure level of 1,600 WLM led to 18% of the rats developing lung cancer. When radon exposure was combined with tobacco smoke, there was a significant increase in the incidence of lung cancer. This pattern was also observed in rats exposed to 1800 WLM of radon and 350 h of tobacco smoke, which exhibited a lung cancer rate twice that of rats exposed only to radon (50). The increase in incidence following joint exposure in rats was estimated to be 2 to 4 times, depending on the cumulative exposure to radon and the duration of exposure to tobacco smoke (30). It should be emphasized that these exposure doses are considerably higher than typical residential radon levels. Nevertheless, these studies illustrate a synergistic effect between tobacco smoke and radon. They also contribute to a better understanding of the observations from the initial cohorts of miners exposed to elevated radon levels. Historically, miners from the 1940s were exposed to annual doses exceeding 400 WLM, which were subsequently reduced to approximately 2 WLM per year by the 1970s and to less than 1 WLM at the beginning of the 2000s (55). Currently, the limits established by the Mine Safety and Health Administration (MSHA) and Occupational Safety and Health Administration (OSHA) are 4 WLM per year (56). However, no studies identified in StoreDB have examined the impact of radon alone or combined with tobacco smoke at doses and dose rates comparable to environmental levels. This indicates a gap in this area, particularly concerning combined exposure, which aligns with previous observations noted in the literature.

**Figure 4:**
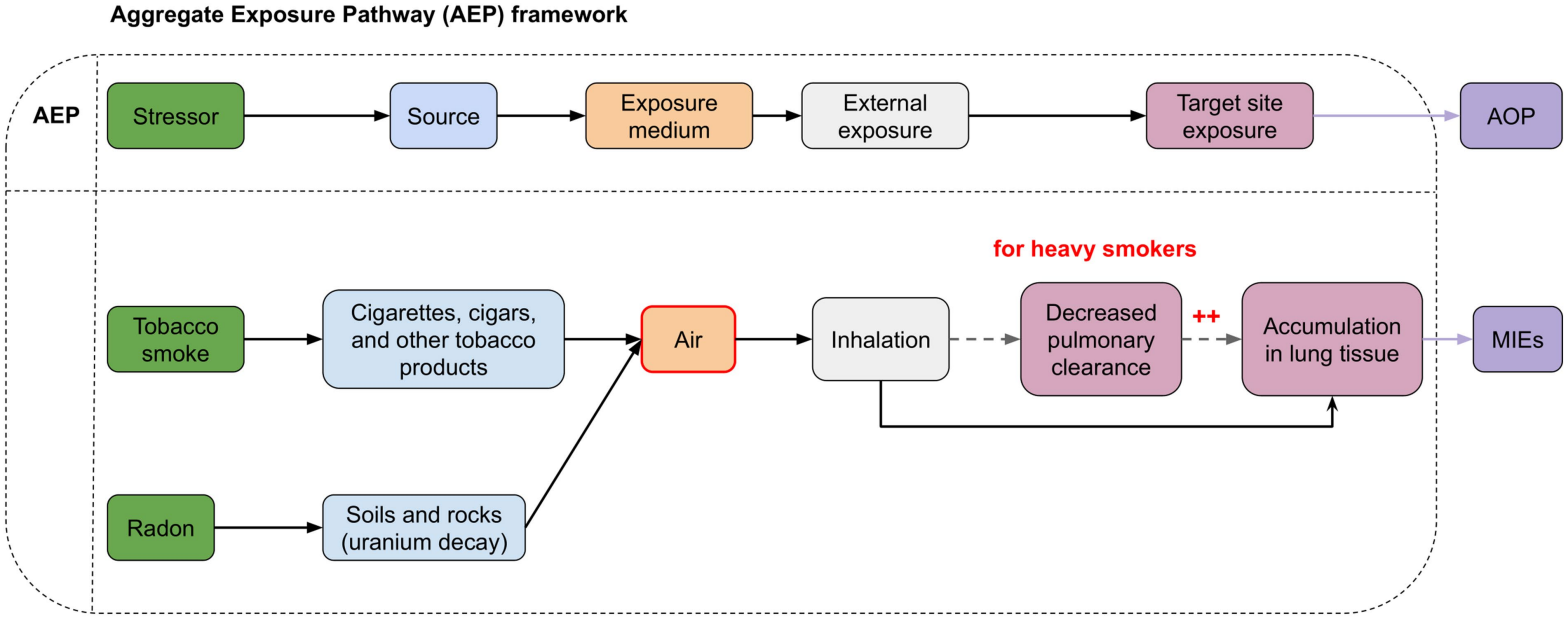
Aggregate Exposure Pathways (AEP) illustrating the interconnected risk arising from simultaneous exposure to tobacco smoke and radon. The red areas indicate points of interaction. Radon decay products can attach to tobacco smoke particles in the air, resulting in higher concentrations of radon (and radon products) in smoking areas. In heavy smokers, changes in lung function lead to reduced elimination of radon and tobacco smoke from the lungs. This results in greater accumulation of radon and smoke particles in lung tissues, increasing the potential for molecular damage.

The interaction observed between these two risk factors can be attributed to different mechanisms, some of which can be explained during the exposure phase, as outlined in the AEP (Figure 4). Indeed, the association can occur in the environment. It has been observed that radon decay products can also attach to tobacco smoke particles in the air, resulting in higher concentrations of radon (and radon products) in smoking areas, thereby amplifying radon exposure (57). Furthermore, heavy smokers show structural and functional changes in the lungs, such as reduced pulmonary clearance, leading to an increased accumulation of both tobacco smoke, radon, and its decay products in the respiratory tract. As a result, the radiation dose from a specific radon concentration is significantly higher in heavy smokers compared to non-smokers (33, 58). For heavy smokers, the radiation dose may be twice that received from the same level of radon exposure in non-smokers (59) (Figure 4). This observation, however, appears to be specific to heavy smokers with decreased mucociliary clearance, reduced lung volume, and increased respiratory rate. In contrast, for lighter smokers, the effect might be inverse, potentially due to a thicker layer of mucus (58, 60).

Thus, the representation of exposure pathways in the form of an AEP illustrates the complex interplay between radon and tobacco smoke and their association from the environment to the exposure site (i.e., lung tissue). The use of such approaches is crucial to understand all the risks associated with this combined exposure in order to formulate comprehensive prevention and control strategies aimed at protecting public health.

### 3.3 AOPN proposition

#### 3.3.1 Underlying biological mechanisms

This AOPN was developed to illustrate the mechanisms shared between lung cancer development induced by both radon and tobacco smoke. It highlights how these stressors disrupt common biological pathways, leading to the disease. The specificity of each stressor for these shared events were carefully considered. Additionally, the distinct natures of these factors were addressed, with separate discussions on the MIEs.

##### 3.3.1.1 Distinct molecular initiating events induce distinct DNA damage

###### 3.3.1.1.1 Activation of carcinogens from tobacco smoke

Many carcinogens in tobacco smoke act as procarcinogens, requiring metabolic activation to exert their genotoxic effects. This includes dioxins, dioxin-like compounds, polycyclic aromatic hydrocarbons (PAHs) such as benzo[a]pyrene (BaP), and nitrosamines like NNK and NNN (61–63). These compounds are bioactivated primarily through cytochrome P450 (CYP) enzymes, often regulated via the aryl hydrocarbon receptor (AHR) pathway. AHR is expressed in multiple tissues, including bronchial epithelial cells, where it modulates signaling pathways implicated in lung tumorigenesis (64). Upon ligand binding, AHR translocates to the nucleus, dimerizes with ARNT, and activates transcription of target genes, including those encoding CYP enzymes. For instance, BaP is metabolically activated to BPDE, a highly reactive metabolite that forms bulky DNA adducts (65, 66). Nitrosamines such as NNK and NNN are activated independently of AHR via CYP2A6 and CYP2A13, and also contribute to DNA adduct formation and carcinogenic processes (3, 67, 68).

###### 3.3.1.1.2 Deposition of ionizing energy from radon

Upon undergoing radioactive decay, radon and its progeny emit IR, primarily in the form of alpha particles and, to a lesser extent, beta particles. Alpha particles composed of two protons and two neutrons are emitted by radon progeny such as polonium 218 and polonium 214. Being highly ionizing, with high linear energy transfer (LET) and a short range, they are recognized as the main contributors to lung tissue dose through the induction of complex DNA damage in the bronchial epithelium, including clustered lesions and double-strand breaks (DSBs) (18, 28, 69, 70). In contrast, beta emissions are also produced, but due to their lower LET and greater penetration depth, they are less biologically effective in damaging bronchial epithelial cells and therefore less significant in terms of lung carcinogenesis.

###### 3.3.1.1.3 Generation of reactive oxygen species

Both tobacco smoke and radon contribute to the formation of reactive oxygen species (ROS), which play a central role in oxidative DNA damage and carcinogenesis. Tobacco smoke contains numerous radical-generating compounds in both its gaseous and particulate phases. The gaseous phase includes nitric oxide and nitrogen dioxide, which can react with superoxide to form peroxynitrite. The particulate phase (tar) contains dihydroxybenzenes (e.g., hydroquinone, catechol), capable of auto-oxidation into semiquinone radicals that further generate ROS such as superoxide anions (71–73). Additionally, the metabolism of tobacco-derived PAHs, such as BaP, via aldo-keto reductases (AKRs), leads to the formation of ROS-producing catechols (74). AKR1C3, in particular, has been shown to be upregulated following exposure to tobacco smoke or radon (75), and a polymorphism in this gene (rs12529) is associated with increased lung cancer risk (76). Radon also induces ROS production through water radiolysis caused by alpha particle emissions, leading to the formation of hydroxyl radicals that can directly attack DNA (18). When ROS production surpasses antioxidant defenses, oxidative DNA damage occurs, including oxidized bases like 8-oxoguanine, which can mispair with adenine and lead to G: C > T: A transversions, as well as single-strand breaks, abasic sites, and bulky lesions (77–81).

Beyond these biochemical mechanisms, interindividual variability in oxidative stress response may further influence vulnerability to lung cancer in the context of radon and tobacco exposure. Several studies have highlighted the role of genetic polymorphisms in key antioxidant and DNA repair genes. For instance, individuals lacking *GSTM1*, an enzyme involved in ROS detoxification, exhibited greater lung cancer risk when exposed to elevated levels of residential radon or secondhand smoke, supporting a gene–environment interaction via oxidative pathways (76). Similarly, the cumulative presence of variants in oxidative stress-related genes such as *GSTM1, MPO, OGG1, TP53,* and *MMP1* was associated with a markedly increased risk of lung cancer in a Thai population, particularly among women, suggesting that inherited deficiencies in ROS-handling mechanisms may exacerbate the effects of environmental exposures like radon and passive smoking (82). These observations underscore the importance of considering both the combined exposure to radon and tobacco smoke, which may amplify their individual effects, and the role of individual susceptibility in modulating the impact of ROS on lung tumorigenesis.

##### 3.3.1.2 Mutations following DNA repair errors/inefficiency

**Figure 5:**
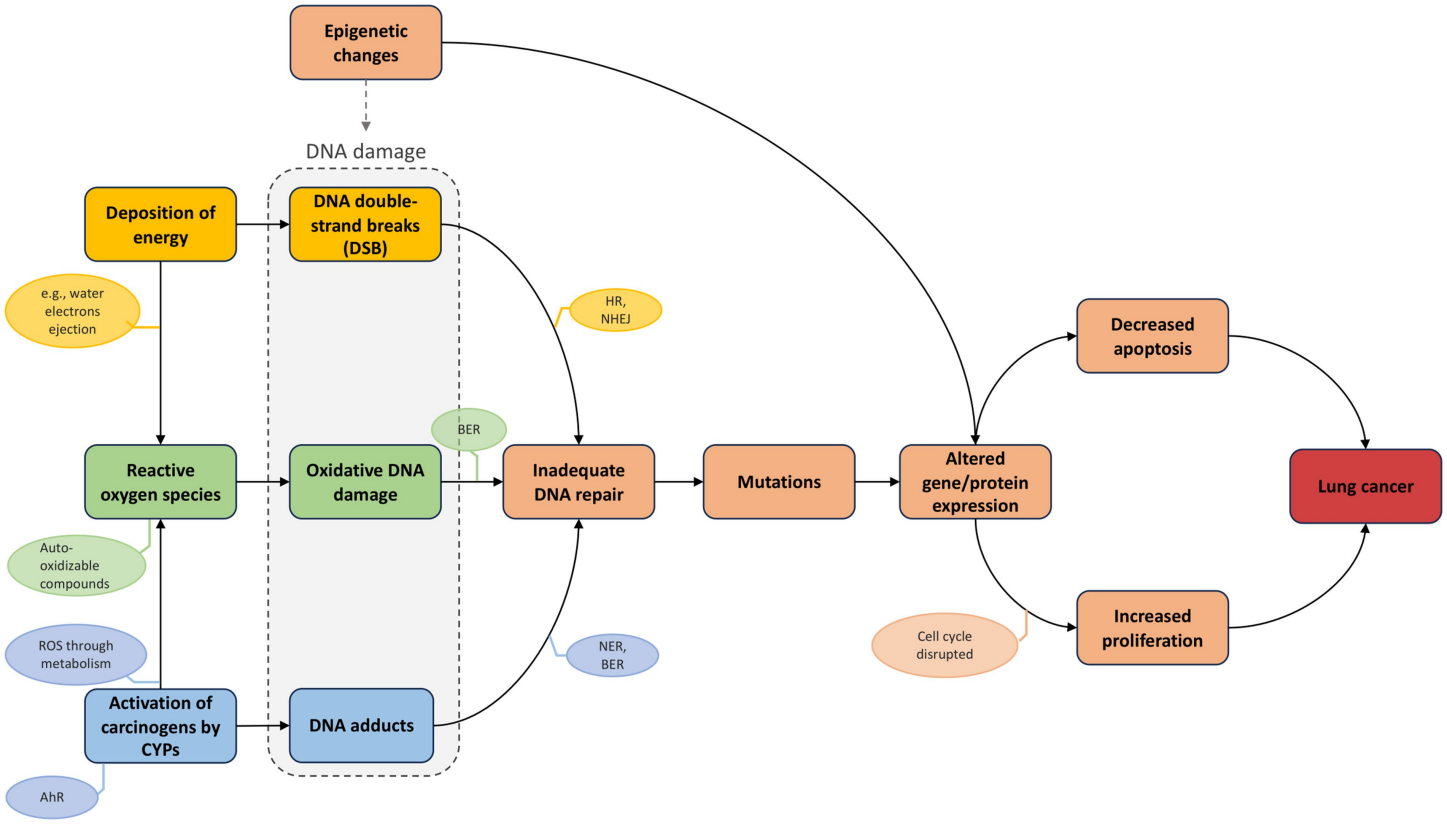
AOPN triggered by combined exposure to radon (physical agent) and tobacco smoke (chemical agent). The AOPN illustrates distinct MIEs initiated by radon (“deposition of energy”), tobacco smoke (“uptake of carcinogens by CYPs”), or both (“Reactive oxygen species”), each leading to different types of DNA damage that converge on a shared pathway via the KE “Inadequate DNA repair.” The AO is labeled as “Lung cancer,” reflecting a generic term encompassing increased risk across multiple subtypes. This terminology is consistent with AOP-Wiki conventions, thereby supporting interoperability and linkage across AOPs.

As described in the previous section, co-exposure to radon and tobacco smoke induces a variety of DNA lesions (Figure 5). These damages are processed by specific repair pathways: nucleotide excision repair (NER) addresses bulky adducts, such as those formed by metabolized PAHs and nitrosamines; base excision repair (BER) corrects oxidative lesions like 8-oxoguanine; and double-strand breaks (DSBs), typically caused by IR from radon, are repaired through non-homologous end joining (NHEJ) or homologous recombination (HR) (34, 79, 83–87). When DNA damage accumulates beyond the capacity of these systems, especially under chronic co-exposure conditions, genomic instability may develop, promoting the accumulation of mutations and chromosomal rearrangements. These repair limitations contribute to mutation accumulation in oncogenes and tumor suppressor genes, with distinct patterns depending on the initiating stressor.

Tobacco smoke is associated with a high frequency of single nucleotide variants (SNVs), notably G: C > T: A transversions caused by BPDE adducts. These are particularly frequent in *KRAS* (especially at codon 12) and in *TP53*, which exhibits characteristic mutations at PAH-binding hotspots such as codons 157, 158, 245, 248, and 273 (88–94). Interestingly, *TP53* mutations have also been observed in uranium miners and residentially exposed populations (95, 96), with a higher prevalence of mutations reported at codon 249 in exon 7. However, the association with specific mutations remains unclear, and further comprehensive analysis of this gene is needed to better understand the mutation spectrum in radon-exposed individuals (96). In contrast, radon-induced DNA damage is more frequently associated with insertions/deletions (indels) and chromosomal rearrangements, consistent with the effects of high LET radiation (97). A higher prevalence of *EGFR* mutations has been reported in areas with elevated radon exposure, with exon 19 deletions showing particularly strong associations. One study found that patients with tumors harboring this deletion had residential radon levels twice as high as those with the L858R point mutation in exon 21, suggesting mutation-specific signatures related to radon (98, 99). Other alterations, such as *ALK* rearrangements and *BRAF* mutations, also appear more frequently in radon-exposed populations and are frequently observed in non-smoking lung adenocarcinomas (97, 98, 100, 101). These distinct mutational patterns likely reflect differences in toxicokinetics, the nature of DNA damage induced, and the repair mechanisms involved—particularly the predominant use of error-prone NHEJ in response to radon-induced DSBs, which promotes deletions and chromosomal rearrangements, while tobacco-related damage more often leads to a mutation landscape dominated by SNVs and transversions due to DNA adducts and oxidative stress (97).

##### 3.3.1.3 Epigenetic changes

In addition to genetic alterations, prolonged exposure to radon and tobacco smoke at environmental concentrations induces epigenetic modifications that contribute to carcinogenesis. These alterations affect both specific tumor suppressor genes and global genomic regulation.

Localized promoter hypermethylation has been observed in bronchial epithelial cells following combined exposure, leading to changes in cell cycle progression (e.g., reduced G1 and increased S phase) (102). This is supported by evidence of hypermethylation of *CDKN2A* and *RASSF1A* in response to radon and tobacco smoke, respectively, two key regulators of cell cycle control and apoptosis (103–108). Tobacco smoke may also influence the epigenetic regulation of xenobiotic metabolism. In lung tissues of smokers, lower methylation levels at the *CYP1A1* enhancer were associated with higher DNA adduct burdens and the presence of *TP53* or *KRAS* mutations in corresponding tumor tissues (109). This suggests that demethylation-driven *CYP1A1* upregulation may increase the bioactivation of tobacco carcinogens, reinforcing the mutagenic pressure.

In parallel, combined exposure also results in global DNA hypomethylation, a hallmark of many cancers (102, 110, 111). This reduction in methylation promotes genomic instability by reactivating transposable elements and altering the expression of normally silenced genomic regions. In addition, changes in histone marks, such as increased acetylation or decreased methylation, lead to chromatin relaxation, thereby increasing DNA accessibility and susceptibility to damage from ROS and IR (112, 113).

Together, these epigenetic alterations, including site-specific methylation changes and global DNA hypomethylation, destabilize the genome, silence tumor suppressor genes, and enhance the metabolic activation of carcinogens. These tightly interconnected processes affect multiple KEs in the AOPN and underscore the need to integrate both genetic and epigenetic mechanisms in the assessment of lung cancer development under combined exposures.

##### 3.3.1.4 Impact of cell cycle alteration and reduced apoptosis on lung cancer

Building on the genomic and epigenomic alterations described above, the disruption of cell cycle checkpoints and apoptotic control constitutes a critical cellular transition in the development of lung cancer. These changes confer a proliferative advantage, allowing damaged cells to bypass key regulatory barriers.

In lung cancer, particularly non-small cell lung cancer (NSCLC), oncogenic alterations such as mutations in *KRAS*, *EGFR*, *BRAF*, and *ALK* rearrangements activate key signaling cascades, most notably the RAS–RAF–MEK–ERK and PI3K-AKT–mTOR pathways. These pathways promote Cyclin D1 expression and CDK4/6 activation, driving the G1/S cell cycle transition, while concurrently inhibiting pro-apoptotic mediators like BAD and BIM. This dual action favors unchecked proliferation and resistance to apoptosis, translating upstream mutational events into downstream malignant phenotypes (114–118). Concomitantly, loss of tumor suppressor function, particularly through *TP53* inactivation, one of the most frequent events in lung cancer, removes critical safeguards. *TP53* coordinates cell cycle arrest and apoptosis in response to cellular stress. Its loss impairs DNA damage checkpoints and apoptotic response, further enabling unchecked division of genomically unstable cells (118–120). Murine studies with specific *TP53* mutants have shown that preserving either apoptotic or cell cycle regulatory functions alone is sufficient to delay tumorigenesis compared to complete *TP53* loss, highlighting the distinct and complementary contributions of these two processes (119, 120). Beyond genetic mutations, epigenetic silencing of genes like *CDKN2A* and *RASSF1A*, already discussed in the previous section, also contributes to this dysregulation. These genes are essential for enforcing cell cycle arrest and promoting apoptotic signaling, and their inactivation via promoter hypermethylation exacerbates the effects of mutational inactivation by disrupting the same regulatory axes (107, 108, 121).

Together, the imbalance between proliferative signals and apoptotic resistance drives the persistence and uncontrolled growth of altered cells. This highlights the functional consequences of earlier molecular lesions and reinforces the pivotal role of “Increased proliferation” and “Decreased apoptosis” as central KEs in lung cancer initiation within the AOPN framework.

##### 3.3.1.5 Histological types of lung cancer

Lung cancer is broadly classified into two main categories: small cell lung cancer (SCLC), which accounts for approximately 15% of cases, and non-small cell lung cancer (NSCLC), comprising the remaining 85% (122). SCLC arises predominantly in the mid-level airways and is characterized by rapid growth, early metastasis, and poor prognosis (123, 124). Genomic analyses have shown that SCLC tumors exhibit extensive chromosomal rearrangements and a high mutation burden, with near-universal inactivation of the tumor suppressor genes *TP53* and *RB1* (125). Strongly associated with tobacco smoking, the incidence of SCLC has declined since the late 1980s, likely due to reductions in smoking prevalence and changes in cigarette composition (122, 126). Radon exposure has also been linked to this subtype: a meta-analysis of 28 case–control studies reported a significantly increased risk of SCLC associated with residential radon exposure (OR = 2.03), particularly among smoker (35). This finding is supported by histopathological reviews of lung cancer cases in uranium miners and atomic bomb survivors, showing a higher frequency of SCLC following radiation exposure (127).

Within NSCLC, adenocarcinoma is the most frequently diagnosed subtype, accounting for around 40% of all lung cancers (128, 129). Arising from mucus-secreting epithelial cells located in the peripheral lung, it is associated with distinct genetic alterations. *KRAS* mutations are common in this subtype, observed in 20–40% of cases, and are more frequently detected in smokers and individuals of Western descent (88, 130, 131). In contrast, *EGFR* mutations, found in approximately 10–15% of patients, are more typical of non-smokers and individuals of Asian origin (131–134). Other genetic alterations such as *ALK* rearrangements, although less frequent, are also predominantly observed in non-smokers or light smokers (131, 135, 136). Interestingly, some of these molecular features, particularly *EGFR* and *ALK* alterations, appear at higher rates in populations residing in radon-prone areas, suggesting potential subtype associations with radon exposure (see Section 3.3.1.2) (97–101). Supporting this, a meta-analysis reported a significantly increased risk of adenocarcinoma in low-smoking populations exposed to residential radon (OR = 1.58) (35).

Squamous cell carcinoma, another major NSCLC subtype, typically originates from the central bronchi and accounts for roughly 25–30% of lung cancers (128, 129). It has long been linked to tobacco smoking, as consistently reported in epidemiological studies (137–139), and is molecularly characterized by a high frequency of *TP53* mutations (140). Though to a lesser extent than for adenocarcinoma or SCLC, a significant association between residential radon exposure and squamous cell carcinoma has also been documented (35). Moreover, a study conducted in women treated with radiotherapy for breast cancer reported that those who smoked at the time of treatment had a significantly increased risk of developing squamous cell carcinoma later on, highlighting a synergistic interaction between tobacco smoke and IR (141).

Thus, while some histological types, such as squamous cell carcinoma, appear more strongly linked to tobacco smoking than to radon exposure, the mechanistic basis for these distinctions remains incompletely understood and is currently under active investigation, including within the RADONORM consortium.^6^ Nevertheless, it is well established that both radon and tobacco smoke contribute to the development of various lung cancer subtypes, including both SCLC and NSCLC. Indeed, although they differ in their toxicokinetic properties and mutational signatures, their ability to reach distinct regions of the lung and induce a wide range of molecular alterations may help explain their association with multiple histological subtypes.

#### 3.3.2 Computational evaluation of KERs

The weight-of-evidence for the KERs described in the AOPN was assessed using a computational, literature-based approach (see Materials and methods, section 2.4.2). Briefly, the *event–event* module of AOP-helpFinder was used to analyze PubMed abstracts in a stressor-agnostic manner, enabling the calculation of a *Cs* for each KER. This score reflects how strongly a given pair of events is correlated and supported in the scientific literature, based on their co-occurrence frequency. It is categorized into five levels, ranging from low to very high confidence. Overall, the AOPN model includes well-supported relationships, with the majority of KERs receiving high or very high *Cs* values. These results are summarized in Table 1, which also reports the number of supporting abstracts found in PubMed for each event pair. To improve scoring accuracy, multiple synonyms were used for each KE (see Supplementary Table S3). In addition to this computational analysis, curated information from the AOP-Wiki was consulted when available to complement the literature-based evaluation performed using AOP-helpFinder. These details are presented below for individual KERs:

- **Deposition of energy leads to DNA double-strand breaks:** This KER is strongly supported both by the literature and the AOP-Wiki. AOP-helpFinder assigned a *very high* Cs to this relationship, identifying over 700 relevant abstracts (KER 1, Table 1). This is consistent with existing entries in the AOP-Wiki, notably KER 1977 (“Energy Deposition leads to Increase, DNA strand breaks”), which is included in six AOPs and extensively describes the mechanistic link and dose–response relationship. This KER is also a key feature of AOP 272, which specifically addresses lung cancer. Additionally, KER 2380 (“Ionizing Energy leads to Increase, DNA Damage”) reinforces this relationship across four other AOPs, further confirming the central role of energy deposition in initiating DNA damage.
- **Activation of carcinogens by CYP leads to DNA adducts:** Although only 25 abstracts were identified linking cytochrome P450 enzymes, particularly CYP1A1 and CYP1B1, to DNA adduct formation (KER 3, Table 1), this relatively low number likely reflects the indirect nature of the relationship. In most studies, CYP activation is described as an upstream event leading to the production of reactive metabolites, which are themselves responsible for DNA adduct formation. Nonetheless, the tool assigned a high *Cs* to this KER, indicating frequent co-mentioning relative to the overall literature on each event. As described in Section 3.3.1.1, this relationship is mechanistically well supported in the context of tobacco smoke, where AHR-mediated induction of CYPs facilitates the metabolic activation of procarcinogens such as PAHs and nitrosamines. This AHR–CYP relationship is comprehensively described in the literature, evidenced by a high Cs (KER 3 – Table 1), and is also detailed in AOP-Wiki (KER 19), where it is supported across several AOPs (e.g., AOPs 494 and 57).
- **Deposition of energy and CYP activation leads to reactive oxygen species:** The relationships between energy deposition and ROS (KER 2) as well as between CYP activation and ROS (KER 4) are well supported, with high *Cs* assigned by AOP-helpFinder (Table 1). These associations are further substantiated in AOP-Wiki. For example, KER 2893 links CYP1A2 to ROS production, while KER 2887 describes the connection between CYP1A1 and ROS, particularly in the context of AHR activation, as detailed in AOP 494. Similarly, KER 1512 describes the role of CYP2E1 in oxidative stress, especially in carcinogenic processes. The link between energy deposition and ROS is also documented in KERs 2,379 and 2,557, notably within AOP 311, which explores how IR promotes ROS generation and subsequent oxidative DNA damage.
- **Reactive oxygen species leads to oxidative DNA damage:** The correlation between ROS and oxidative DNA damage (KER 5) is strongly supported in the literature, with nearly 1,200 PubMed abstracts identified by AOP-helpFinder and a very high *Cs* assigned (Table 1). This relationship is further reinforced in AOP-Wiki through multiple KERs. For instance, KER 2590, featured in AOPs 299 and 311 (both triggered by IR), and KER 2099 consistently describe the link between increased ROS and oxidative DNA damage with high confidence. Additionally, KER 2810 in AOP 478 connects oxidative stress to oxidative DNA damage, while KER 2811 links oxidative stress to DNA strand breaks in AOPs 470, 478, and 483, all initiated by IR. Finally, KER 1300, used in AOP 200, describes the broader relationship between oxidative stress and DNA damage in the context of breast cancer.
- **DNA damage leads to inadequate DNA repair and mutations:** DNA damage is typically repaired before replication, but when lesions persist or repair is faulty, as can occur via error-prone pathways like NHEJ, mutagenesis may ensue, contributing to carcinogenesis through the accumulation of alterations in key genes, as detailed in Section 3.3.1.2. In the AOP-helpFinder analysis, the link between DNA damage and inadequate DNA repair (KER 6) was supported by 36 abstracts and a moderate *Cs*. This relatively modest result likely stems from the limited use of specific terminology such as “inadequate DNA repair” in PubMed-indexed abstracts. A similar trend was observed for the connection between inadequate repair and mutations (KER 7), with 68 articles retrieved. However, the broader link between DNA damage and mutations was strongly supported, with nearly 4,000 articles identified and a very high *Cs* (KER 8 – Table 1). These relationships are also well documented in AOP-Wiki: for instance, AOP 397 highlights the progression from bulky DNA adducts to mutations through inefficient repair. Additional KERs, such as KER 1909 (oxidative DNA damage leads to inadequate repair), KER 164 (inadequate repair leads to mutations), and KERs 1899, 1914, 1931, and 2,399 further describe the connections between specific types of DNA lesions and mutagenesis across multiple AOPs, including those related to lung carcinogenesis. Collectively, these data underscore the critical role of DNA repair fidelity in preventing mutation accumulation and genomic instability.
- **Mutations and epigenetic changes lead to altered gene and protein expression/function:** The link between mutations and altered gene expression is well supported, as reflected by the high *Cs* and the 1772 abstracts identified by AOP-helpFinder for this KER (KER 8 – Table 1). This relationship is also described in AOP-Wiki under KER 1301, “Increase, DNA damage leads to altered, gene expression,” particularly in the context of cancer.
  Similarly, the association between epigenetic changes and altered gene expression is strongly documented, with over 500 supporting abstracts and a high Cs assigned to KER 9 (Table 1). AOP-Wiki KER 1884 further details this link, specifically connecting DNA hypomethylation to increased gene expression. As discussed in Section 3.3.1.3, epigenetic alterations, especially global hypomethylation, can also promote genomic instability and DNA damage (non-adjacent KER with high Cs; Table 1), underscoring their multifaceted contribution to carcinogenesis.
- **Increased cell proliferation and decreased apoptosis, resulting from altered gene expression, lead to lung cancer:** The roles of increased cell proliferation and reduced apoptosis in carcinogenesis are well established in the literature, as reflected by the high *Cs* values and substantial number of articles retrieved by AOP-helpFinder for these KERs (KERs 10, 11, 12 – Table 1). Within AOP-Wiki, KER 1978 captures the relationship between “Increased mutations” and “Increased cell proliferation”, with strong supporting evidence in the context of lung cancer, particularly in AOP 272. The link between proliferation and tumorigenesis is further supported by KERs such as 1967 (“Increased cell proliferation leads to lung cancer”) and 1980 (“Increased cell proliferation leads to an increase in lung cancer”). In AOP-Wiki, reduced apoptosis is generally categorized under the broader KE “Increased cell proliferation” to facilitate KER evaluation, as observed in AOP 272. However, in this AOPN, these two processes are considered separately to reflect their distinct biological contributions. As detailed in Section 3.3.1.4, studies on *TP53* mutants have shown that either preserved apoptotic function or intact cell cycle control alone can delay tumorigenesis, emphasizing the independent importance of each mechanism. These cellular-level disruptions represent a functional consequence of prior genetic and epigenetic alterations and serve as pivotal KEs in the transition from molecular damage to malignant transformation.

### 3.4 Connection of the AOPN with other existing lung cancer AOPs

To date, six AOPs related to lung cancer are referenced in AOP-Wiki (AOP 416, 417, 420, 272, 303, and 451). Our AOPN stands out by addressing both genetic and epigenetic aspects of lung cancer while integrating combined exposure to chemical and physical factors. Additionally, our AOPN can be synergistically combined with existing AOPs to offer a more comprehensive perspective on lung cancer progression.

AOPs 416, 417, and 420 underscore the significance of AHR activation in this malignancy. AOP 417 incorporates elements discussed in the AOPN through the production of active metabolites that can cause DNA damage following AHR activation (e.g., bioactivation of PAHs by CYPs) (64). On the other hand, AOP 416 highlights an alternate mechanism of AHR activation, distinct from the AHR/ARNT interaction. Research has shown that AHR can bind with other proteins, thereby influencing their activity. Overexpression of AHR leads to increased nuclear translocation of RELA (p65) and the formation of an AHR/RELA complex. This complex subsequently associates with an κB element (p50), augmenting NF-κB activity. This dynamic results in an upregulation of Interleukin-6 (IL-6), a pivotal molecule in lung tumor initiation (64). AOP 420 delineates the sustained activation of the NRF2 pathway following the activation of the AHR. NRF2 is activated by oxidative stimuli, especially those generated by tobacco smoke components. Once activated, NRF2 predominantly acts as a tumor suppressor, orchestrating various biological pathways. It counters oxidative stress by associating with antioxidant response elements (ARE) and triggering the expression of antioxidant and detoxifying genes (142). However, some studies suggest that hyperactivation of NRF2 might exhibit pro-oncogenic characteristics in the context of lung cancer (64, 143). Specifically, sustained NRF2 activation has been linked to increased survival of tumor cells, attributed to NRF2’s role in regulating cell proliferation and differentiation (143). Therefore, these three AOPs complement our AOPN by addressing KEs related to oxidative stress, DNA damage and increased cell proliferation induced by tobacco smoke exposure. They deepen the understanding of smoking’s impact on the trajectory towards lung cancer, spotlighting the diverse roles of AHR in this disease’s development.

Conversely, AOP 272 focuses on the effects of IR on lung cancer initiation. This AOP identifies the deposition of ionizing energy as the MIE, leading to DSB and subsequent mutations due to the errors in DNA repair mechanisms. These genetic abnormalities are likely to promote progression to lung cancer. Many aspects of AOP 272 overlap with and complement those of our own AOP. This AOP is endorsed by the OECD, conferring increased recognition and credibility to its relevance in the context of radiation-induced lung cancer, thereby attesting to the validity of the pathway described. It also highlights certain uncertainties associated with the AOP, particularly concerning the quantitative and essentiality studies (18). This corroborates our previous observations pointing to a lack of mechanistic data, in contrast with the abundancy of cohort studies and case–control studies.

Additionally, AOP 451 highlights the impact of various genotoxic agents such as nanoparticles or diesel engine emissions, among others. It specifically focuses on the interaction of these agents with the cell membrane components of lung cells. These persistent agents induce chronic inflammation and oxidative stress, identified as the primary KEs (144). Similarly, AOP 303 examines the impact of High Aspect Ratio Materials (HARMs) on lung cancer progression, highlighting ROS and inflammation. HARMs notably include materials such as asbestos fibers, which can still be found in older buildings. Both AOP 451 and AOP 303 align with the points outlined in our AOPN and highlight additional stressors from our daily exposome, such as diesel engine emissions, prevalent in urban environments.

When integrated, the collective observations from these six AOPs not only refine our specific AOPN but also expand its relevance by incorporating additional stressors (e.g., air pollutants or occupational exposures) and KE (e.g., inflammation) not addressed in the present study. While initially focused on co-exposure to radon and tobacco smoke, this AOPN structure provides a flexible framework to capture broader mechanisms involved in lung carcinogenesis. As more AOPs become available, describing pathways triggered by diverse agents, the network can be progressively enriched to reflect the full complexity of real-world exposures.

## 4 Discussion

This study illustrates the potential and utility of computational tools based on AI and machine learning in developing alternative toxicological models like AOPs, AEPs, and AOPNs. The PubMed database currently catalogs over 36 million scientific articles, within which relevant information can be widely scattered and difficult to retrieve. Tools such as AOP-helpFinder and PubTator used in this research enabled the rapid extraction of a substantial corpus of articles on radon, tobacco smoke, and lung cancer, greatly assisting in evaluating and interconnecting existing knowledge. This facilitated the proposition of integrative models based on AEPs and AOPs, highlighting complex interactions from environmental exposures to molecular and cellular mechanisms. The literature search strategy relied on keyword-based queries targeting KEs and the AO of lung cancer, using terms such as “small cell lung cancer,” “non-small cell lung cancer,” “adenocarcinoma,” and “squamous cell carcinoma,” rather than relying on ICD or DSM codes. These were combined with exposure-related terms (e.g., radon, tobacco smoke) to identify relevant publications. Nevertheless, AOP-helpFinder operates based on exact keyword matching, which inherently limits its ability to capture all relevant literature when terminologies vary. To address this, we compiled an extensive list of 238 biological events related to lung cancer from expert databases such as AOP-Wiki, CTD, GeneCards, and DisGeNET (Supplementary Table S1), in order to maximize retrieval across heterogeneous reporting styles. Despite this effort, it is likely that some relevant articles were missed, potentially resulting in false negatives, meaning that the total number of studies addressing radon and tobacco co-exposure in lung cancer may exceed the 378 publications identified. These 378 shared articles were subsequently clustered based on information extracted from the texts, helping prioritize sources for the construction of the AEP and AOPN models (see section 2.2). Although this clustering approach was effective, it did not allow for automatic histology-specific separation due to heterogeneity in data reporting. This limitation is acknowledged, and future iterations of the framework may incorporate histology-stratified analyses as more structured and mechanistically detailed data become available.

From the onset, the AEP highlighted an environmental interaction between radon and tobacco smoke through the attachment of radon decay products to tobacco smoke particles in the air, resulting in higher concentrations of radon (and its decay products) in smoking areas. Moreover, in active smokers, a chronic inflammation takes root in the lungs and plays a pivotal role in lung cancer initiation by compromising pulmonary function. For instance, smoking-related pulmonary disorders, such as chronic obstructive pulmonary disease (COPD), correlate with an abnormal release of inflammatory cytokines. These disruptions in both inflammatory and fibrotic pathways, typical of COPD, set the stage for the epithelial-mesenchymal transition (EMT) (145). More specifically, smoke triggers a neutrophilic inflammation in the bronchial epithelium, which, in turn, diminishes mucociliary clearance in the lungs as detailed in the AEP. This reduced clearance mechanism allows for even greater accumulation of radon and tobacco smoke products, leading to increased DNA damage (146). Furthermore, a study revealed an alteration in cytokine profiles among miners exposed to low doses of radiation over the long term. Another research on uranium miners unveiled an association between a specific *IL-6* promoter polymorphism (rs1800797) and lung cancer (147). This underlines the hypothesis that inflammation, especially when involving pro-inflammatory cytokines, also plays a central role in the onset of lung cancer due to exposure to IR (147, 148). Despite evidence showing the role of inflammation in lung cancer development (e.g., AOPs 416, 451 and 303), it was decided not to directly incorporate this pathway into this AOPN triggered by both radon and tobacco smoke. Indeed, the current evidence does not provide a conclusive picture of the synergistic roles of these two agents on the entire inflammatory process. Instead, it is discussed in the AEP as an exacerbating factor for the AOP, particularly due to the higher accumulation of stressors from physiological changes observed in smokers. However, the role of the immune system, particularly the involvement of the *IL-6* promoter in radon exposure, may be considered as a future perspective and could potentially lead to the development of a specific AOP on lung cancer.

At present, out of the 469 AOPs referenced in the AOP-Wiki database, the majority focus on chemical exposure, though there is an increasing effort in the development of AOPs in the field of IR. As of now, 18 of these AOPs listed on AOP-Wiki are initiated by exposure to IR. Such initiatives provide a more comprehensive view of the damage induced by our exposome and facilitate future collaborations between the radiation domain and other fields. Our AOPN aligns with this approach as it is the first to consider pathways concurrently triggered by a chemical exposure (tobacco smoke) and a physical exposure (radiations emitted by radon). The AOPN also highlights the different lung cancer signatures associated with radon and tobacco smoke. This distinction emerges at the MIE level, leading to distinct types of DNA damage, predominantly adducts from tobacco smoke and DSBs from radon-derived alpha particles (Figure 5), resulting in different mutation patterns. For instance, mutation hotspots in the *TP53* gene have been associated with tobacco smoke (e.g., codons 57, 158, 245, 248, 249, and 273) (93), whereas no such pattern has been clearly established for radon. While biomarkers such as DNA adducts and *TP53* transversions are well characterized for tobacco exposure, potential biomarkers for radon remain largely hypothetical and require further investigation. Nevertheless, despite the differences linked to the nature of these stressors, combined exposure likely contributes to an overload of the DNA repair machinery, thereby increasing the overall risk of mutations. To our knowledge, the proposed AOPN is the first to integrate both genetic and epigenetic alterations in the context of lung cancer. These alterations, including key gene mutations and changes in DNA methylation, may serve as candidate biomarkers for early detection or risk assessment in combined exposure scenarios. This mechanistic framework may also help explain the sub-multiplicative association observed between radon and tobacco smoke (33). Additionally, our AOPN connects with all existing lung cancer AOPs, particularly through KEs related to oxidative stress and DNA damage, thereby enriching current model with both radiation-induced mechanisms (18) and tobacco smoke–associated pathways, including the inflammation process. It also provides a flexible structure that could be extended to incorporate other environmental stressors relevant to lung carcinogenesis.

Concurrently, this computational approach has provided a comprehensive overview of the literature, revealing a relative scarcity of studies on the joint exposure to radon and tobacco smoke, particularly at the molecular and cellular levels. Notably, few studies have investigated the combined impact of these stressors on inflammation or epigenetic mechanisms, both of which warrant further exploration. Additionally, many *in vivo* and *in vitro* studies involve exposure to radon at doses significantly higher than those encountered in residential environments, highlighting a gap in the study of low-dose effects. This issue, also emphasized in AOP 272 “Deposition of energy leading to lung cancer,” reflects both inconsistencies across available studies and the lack of coherent data for quantitative interpretation of radon related outcomes (18). However, in line with the AOP framework, these high-dose studies remain valuable for identifying key biological events and guiding further research at environmentally relevant exposures. This principle is consistent with the radiation protection approach based on the linear no-threshold (LNT) model for cancer risk (149).

There are also uncertainties related to various factors influencing lung cancer risk. For example, while epidemiological studies provide information on the increased risk and incidence of lung cancer following exposure to radon and tobacco smoke, other unstudied stressors (physical, chemical, or even social) can influence the outcomes of these studies, as can the data collection methodology (150, 151). Furthermore, in the context of joint exposure to these two stressors, the era of the research also plays a significant role, leading to uncertainties. For instance, early uranium miners were exposed to higher doses of IR than more recent miners, making comparisons challenging (55). The introduction of cigarette filters has also changed smoking habits and exposure to various toxic compounds, resulting in increased incidence of adenocarcinoma compared to squamous cell cancer or SCLC (126, 152, 153).

Another critical factor to consider is the role of genetic predisposition as a “modifier factor” for radon and smoking. Recent studies have identified pathogenic germline variants (PGVs) in patients with NSCLC, providing new insights into how genetic predisposition may modify the effects of these environmental stressors. For example, variants in genes related to DNA damage and repair or in signaling pathways can increase susceptibility to lung cancer in individuals exposed to radon and tobacco smoke (154–156).

The integrative framework developed in this study may also contribute to informing public health strategies, particularly in residential scenarios where individuals are simultaneously exposed to radon and tobacco smoke. By linking exposures to AOs through defined KEs, the AEP and AOP frameworks enhance transparency and scientific rigor in risk assessment. This is especially relevant given ongoing discussions around dose coefficients, interactions between radon and chemical carcinogens, and the challenges of low-dose extrapolation. Additionally, this mechanistic approach may help bridge the gap between high-dose experimental data and environmentally relevant conditions, and support the identification of early biomarkers for use in risk stratification or targeted prevention, especially among high-risk populations. Altogether, these considerations reflect the complexity of studying joint exposure to radon and tobacco smoke, and this study underscores the need to pursue further efforts in understanding their combined effects to advance both mechanistic insight and public health protection.

## 5 Conclusion

In conclusion, the proposed computational AEP and AOP-based models compile and consolidate the mechanistic connections identified to date, while also highlighting knowledge gaps related to joint exposures to radon and tobacco smoke. This approach demonstrates the potential of AI-driven tools to extract and structure dispersed information from the literature into conceptual frameworks. In the present work, this strategy enabled us to identify and organize existing knowledge on radon–tobacco interactions in the context of lung cancer, providing a foundation for future research and facilitating integration with other stressors and components of the exposome. However, this remains a preliminary and exploratory effort based on automated literature mining. The *Cs* used to assess the weight-of-evidence of each KER offers a structured and reproducible measure based on co-occurrence in PubMed abstracts, but it does not integrate the qualitative assessment criteria provided by traditional frameworks such as the BH criteria. It does not consider study design, data quality, reproducibility, or conflicting evidence. As a result, some relationships may be over- or underestimated due to the computational evaluation being based on publication frequency rather than biological relevance. Moreover, no formal weighting of individual studies was applied, and all data were considered equally regardless of evidentiary strength. While this method enables a high-throughput screening of potential connections, it should be seen as a starting point. Future work should include expert-driven evaluations of the identified KERs using the BH criteria, covering aspects such as biological plausibility, essentiality, empirical support (e.g., dose–response and temporal patterns), and overall coherence across studies. This would allow the refinement and prioritization of the most robust links and strengthen the overall reliability of the proposed network. As such, this work supports the use of integrative, literature-based approaches to guide the development of exposure–effect models and encourages interdisciplinary collaboration. It offers a valuable foundation for future mechanistic investigations and contributes to the broader effort to better characterize the effects of combined environmental exposures.

## Data Availability

The original contributions presented in the study are included in the article/Supplementary material, further inquiries can be directed to the corresponding authors.

## Footnotes

^1^ https://aopwiki.org/

^2^ https://aopwiki.org/

^3^ https://ctdbase.org/

^4^ https://www.genecards.org/

^5^ https://www.disgenet.org/

^6^ https://www.radonorm.eu/
