# Supplementary material for: Comprehensive computational analysis via Adverse Outcome Pathways and Aggregate Exposure Pathways in exploring synergistic effects from radon and tobacco smoke on lung cancer
Source: Front Public Health. 2025 Jul 31;13:1571290. doi: 10.3389/fpubh.2025.1571290 (PMC12350471; doi:10.3389/fpubh.2025.1571290)
Supplement: Supplementary file 1 [file Data_Sheet_1.docx]

Supplementary Material

Comprehensive computational analysis via Adverse Outcome Pathways and Aggregate Exposure Pathways in exploring synergistic effects from radon and tobacco smoke on lung cancer.

Thomas Jaylet^1^, Vinita Chauhan^2^, Laura Mezquita^3^, Nadia Boroumand^4^, Olivier Laurent^5^, Karine Elihn^6^, Lovisa Lundholm^4^, Olivier Armant^7*^, Karine Audouze^1*^

*** Correspondence:**Karine Audouze; [karine.audouze@u-paris.fr](mailto:karine.audouze@u-paris.fr)
Olivier Armant; olivier.armant@asnr.fr

**Figure S1: Elbow curve to determine the optimal number of clusters for the clustering of the 378 annotated articles related to the association between radon, tobacco, and lung cancer.** Based on the analysis of the figure, four clusters were chosen.

**Table S1: List of 282 biological events (MIEs, KEs, and AOs) involved in or related to lung cancer, extracted from various databases.** For each event, the database from which it was extracted is noted. This list was subsequently used as input for the 'stressor-event' searches in AOP-helpFinder.

**Table S2: List of the 378 articles related to the association between radon, tobacco, and lung cancer.** For each article, the table contains various descriptors: events identified by AOP-helpFinder, event category (MIE, KE, AO), information retrieved by PubTator (studied model or species: human, animal models, or cellular models), as well as the cluster number assigned by t-SNE.

**Table S3: Computational KERs evaluation using AOP-helpFinder.** Supplementary table to Table 1, showing for each KE the different synonyms searched by AOP-helpFinder to maximize the identification of articles describing KERs. The synonyms for each KE are separated by a pipe ( | ). The stars represent the Cs between the evaluated links by AOP-helpFinder. [★★★★★] corresponds to a “Very High” Cs, [★★★★] to a “High” Cs, and [★★★] to a “Moderate” Cs. The Cs [★★], [★], corresponding to "Quite Low" and "Low", respectively, were not found. The 'Link' column corresponds to the number of links from PubMed abstracts found between each pair of KEs.

**Figure S1: Elbow curve to determine the optimal number of clusters for the clustering of the 378 annotated articles related to the association between radon, tobacco, and lung cancer.** Based on the analysis of the figure, four clusters were chosen.

**All supplementary tables can be found at:** [**https://zenodo.org/records/13895866**](https://zenodo.org/records/13895866)
